# Relative contributions of urine sulfate, titratable urine anion, and GI anion to net acid load and effects of age

**DOI:** 10.14814/phy2.14870

**Published:** 2021-05-27

**Authors:** Jenny Huo, Daniel Li, Charles McKay, Madeleine Hoke, Elaine Worcester, Fredric Coe

**Affiliations:** ^1^ Department of Medicine University of Chicago Medicine Chicago Illinois USA

**Keywords:** acid production, ammonia, net acid excretion, urinary acidification, urine pH

## Abstract

Models of acid–base balance include acid production from (1) oxidation of sulfur atoms on amino acids and (2) metabolically produced organic acid anions. Acid load is balanced by alkali from metabolism of GI anions; thus, net acid production is equivalent to the sum of urine sulfate and organic anion (measured by titration in urine), minus GI anion. However, the relative contributions of these three sources of acid production in people eating free choice diets, and presumably in acid–base balance, have not been well studied. We collected 26 urines from 18 normal subjects (10 male) and 43 urine samples from 34 stone formers (17 male) and measured sulfate, organic anion, and components of GI anion and acid excretion in each; values were expressed as mEq/mmol creatinine. Mean values of the urine components, except creatinine and pH, did not differ between the sexes or groups. Urine organic acid and acid production varied directly with age (*p* ≤ 0.03). In a general linear model of acid excretion, the coefficients for sulfate, organic anion, and GI anion were 0.34 ± 0.09, 0.49 ± 0.12, and −0.51 ± 0.06, respectively, *p* ≤ 0.005, and the model accounted for 54% of the variance. A model for urine ammonia gave similar results. Urine organic anion is a significant contributor to total acid production and may be responsible for an increase in acid production with age.

## Graphical abstract

The kidney must maintain acid‐base balance and acid load from food accounts for only part of daily acid excretion. An equal share comes from metabolic production, which appears to increase with age, while gut alkali absorption neutralizes a variable portion of the acid load. Total acid excretion is best represented by a function that includes all three components.

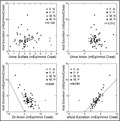

## 1 INTRODUCTION

Systemic acid load and renal acid excretion (AE) are of high importance in the study and care of patients with kidney stones, in large part because urine pH controls urine supersaturation with respect to calcium phosphate and uric acid and therefore the type of stones formed (Coe et al., 2005). Recently, we found that, compared to men on the same diet, women absorb increased amounts of potential alkali as food anions that raise urine pH and predispose them to calcium phosphate stones (Worcester et al., 2018). These anions are estimated from the difference between strongly ionized, non‐metabolizable anions and cations in urine and are commonly called “GI anion” (Oh, 1989). They are assumed to be metabolized in their protonated forms, thus acting to create bicarbonate as they take up protons.

Age affects acid–base handling among stone formers so their urine pH falls (Menezes et al., 2019) and pH‐dependent stone type shifts from phosphate to uric acid (Lieske et al., 2014). What causes the fall in pH is unknown as increased body mass index (BMI) and reduced kidney function were excluded (Menezes et al., 2019), and GI anion excretion actually increases with age, while AE falls.

Contemporary models of acid–base balance include acid production from (Adams et al., (1979)) oxidation of sulfur atoms on methionine and cysteine and (Berg et al., 2020) metabolically produced acid anions that enter the circulation with their protons and impose an acid load (Hamm et al., 2015), which are measured by urine titration (Van Slyke & Palmer, 1920). Acid load is balanced by alkali from metabolism of GI anions; thus, net acid production is equivalent to the sum of urine sulfate and titrated urine organic anion, minus GI anion. Renal AE is taken as the sum of urine titratable acidity (TA) and ammonia, minus urine total CO_2_ content (TCO_2_).

The relative effects of each of these parameters of acid production on AE are unclear. In a crucial balance study using a diet devoid of GI anion (Relman et al., 1961), acid production calculated from the sum of urine sulfate and titrated urine organic anion equaled net renal AE. Evidence from similar studies shows that acid production and AE thus calculated are approximately equal when diet is stable for an appropriate period (Lemann et al., 2003).

A crucial underlying assumption of the modern acid–base model seems rather implausible except perhaps under highly unusual conditions. It is that diet anions estimated from GI anion are all metabolized to produce alkali and that titrated urine organic anions all are produced as acids. These two assumptions are, in fact, mutually dependent. Diet anions that escape metabolism will be titrated as urine anion though not an acid.

This is true because calculated GI anion, titrated urine organic anion, and sulfate all are determined in a single urine aliquot, so conservation must hold in a strict form. GI anion calculation must overestimate alkali to the extent that diet anions are not metabolized, and urine titrated organic anion must include unmetabolized anions which will be accounted as metabolic acid production.

Consequently, the net difference between GI anion and the sum of urine sulfate + titrated organic anion must be constant whatever the true fraction of diet anions is metabolized. Therefore, net acid production can match renal AE, if balance has been achieved experimentally, even though calculated GI anion does not reflect actual alkali production, nor titrated organic anion reflect actual acid production. If indeed some specific group of patients differs from normal, as an example, in the fraction of GI anion converted to alkali, or differ in the amounts of metabolic acids produced, those differences cannot be discovered.

Our purpose here is to test the hypothesis that these three components of acid load are indeed acting as acids or alkali such that 1 mEq of sulfate or anion is 1 mEq of acid, or 1 mEq of GI anion a mEq of base. Our approach was to use renal AE as a reporter function and determine the individual regression slopes of AE on each component of acid production separately and when all three are combined using multivariable general linear regression models.

We also make the same analyses using the components of AE (urine ammonia and TA) and pH separately as dependent variables, in hopes of discovering hitherto unsuspected differences in how GI anion, urine organic anion, and sulfate may affect AE.

## 2 METHODS

### 2.1 Subjects

Unselected new and return patients from our kidney stone prevention clinic provided 24‐h urine collections in the course of their clinical care; in addition, we recruited normal subjects who collected 24‐h urine samples only for this work (institutional review board [IRB] protocols 19–1002, 11943A encompass all participants). We planned no comparisons between patients and normal subjects, nor between the sexes. Our desire was to amass a group of patient and normal urine samples from men and women to use for our analyses. We did not have blood samples from normal subjects, but inspection of serum measurements from the patients revealed no abnormalities of our usual serum measurements: calcium, phosphate, creatinine, uric acid, sodium, potassium, bicarbonate, or chloride.

### 2.2 Routine measurements

Urine sodium, potassium, calcium, magnesium, chloride, phosphate, ammonium, sulfate, creatinine, pH, and uric acid were measured as in our prior publications (Parks et al., 2009). Because patient and normal urines were collected via our usual workflow conditions, they were not under oil; accordingly, we could not reliably estimate bicarbonate. Therefore, we report AE (the sum of ammonia and TA), rather than net AE that would include the contribution of urine bicarbonate, which in most cases would be negligible.

### 2.3 Urine titration

We performed our urine organic anion titrations using methods first reported by Peters and Van Slyke (Van Slyke & Palmer, 1920). We transferred 50 ml of urine into 100‐ml flat‐bottom centrifuge tubes. To these, we added 1 g of calcium hydroxide powder, inverted the centrifuge tubes five to six times, and agitated them at 900 revolutions/minute, for 30 min. We centrifuged the tubes at 3000 × g for 10 min and transferred 40.0 ml of the supernatant to 100‐ml titration vessels. We measured the phosphate concentration of the decanted urine to assure all were below 0.1 mmol/L.

To avoid excessive volume in the titration vessel, when urine ammonia was above 200 µM/L, we added 300–800 μl of 6‐N HCl before titrating the samples down to pH 2.7 using 0.1‐N HCl. We titrated from pH 2.7 to pH 7.4 using 0.1‐N NaOH with a Mettler Toledo G10S titrator (Mettler‐Toledo, LLC). For each run, we established the exact concentration of NaOH by titrating a 0.25‐M potassium phthalate standard made in our lab. Each standard was made using rigorous quantitative techniques, so the exact molarity was known to four significant digits. The titration system was calibrated for each run at five pH points using commercial buffers at pH 2, pH 4, pH 7, pH 10, and pH 13. The final values were corrected for titrated sulfate and for creatinine. Titrated urine sulfate at pH 2.7 is 16.6% of total sulfate given its pK_2_ of 2.01. Therefore, we calculated urine organic anion as mmol/L NaOH consumed between pH 2.7 and pH 7.4 minus 0.166 * measured urine sulfate in mEq/L. Urine creatinine is titrated fully at pH 2.7, and so its concentration was subtracted from the total urine organic anion. Each sample was assayed in triplicate.

### 2.4 Calculations

#### 2.4.1 Definitions

GI anion was calculated as ∑ Na, K, 2 * Ca, 2 * Mg − ∑ Cl, 1.8 * PO_4_ where all values are mEq/L. Valence of phosphate is that at blood pH of 7.4 (Oh, 1989). TA was calculated as the sum of the individual values for titration of urine phosphate, creatinine, and urate from urine pH to blood pH 7.4. AE was calculated conventionally as urine ammonia + TA (mEq/L). Acid production was calculated as (urine sulfate + titrated urine anion) − GI anion (mEq/L).

#### 2.4.2 Normalization for urine creatinine

Usually, one presents acid loads and excretions in mEq/unit time by multiplying concentrations within a urine sample by the urine flow rate. Our question here, however, concerns only the relationships between urine AE and its components urine ammonia, TA, and pH, versus urine organic anion, urine GI anion, and urine sulfate. Multiplication of each value by volume flow can add nothing to such an analysis and adds uncertainties arising from completeness of 24‐h urine collections.

GI anion, urine organic anion, and TA were first calculated in mEq/L, and then the result was divided by urine creatinine in mmol/L to normalize for variations in urine concentration. Urine ammonia and sulfate, being simply measured values, were likewise normalized. It is conventional to express all components of the urine metabolome in this manner (Bouatra et al., 2013), and we wished to make our work compatible with that convention.

### 2.5 Statistical analysis

Means and significance of differences between groups were calculated using routine ANOVA with age as a covariate and subject type, and sex as categorical variables. Because we did not design this research with the aim of identifying group differences, we present only the *p* value for the model itself—whether or not the four groups (subject type and sex) differ among themselves but not *p* values for specific comparisons (e.g., male vs. female patients). The significance of age was estimated and is presented.

Each general linear model (GLM) was of the form:

y=constant+sex+subject type+age+titrated anion+GI anion+sulfate

where *y* was urine AE, ammonia, TA, pH, and phosphate as dependent variables, all expressed as mEq/mmol urine creatinine.

To assure unbiased variable selection, we employed automatic backward stepping to select those variables significant for the final models. We confirmed each model by forward stepping. The software steps independent variables in or out depending on the degree of its correlation with the dependent variable (*p* value in the regression), its tolerance (lack of covariation with other independent variables), and mathematical indices of the information content of the model.

We used Pearson correlation matrices to calculate the individual correlations.

For certain graphical displays, we calculated values for estimated AE, ammonia, TA, and urine pH using coefficients from the GLMs. In calculations of estimates, we used all four decimal places to avoid rounding errors, although we display only two significant digits in tabular reports.

ANOVA, GLMs, and correlation coefficients were produced using standard software (Systat Software Inc.).

## 3 RESULTS

### 3.1 Subjects

We obtained a total of 69 samples from 52 subjects on free choice diets (Table 1). Mean values of the components used in our analysis did not differ between the sexes or groups with the exceptions of age, urine creatinine concentration, and urine pH. Age was used as a covariate in all ANOVA models apart from that for age itself, but only urine organic anion and acid production varied directly with age.

**TABLE 1:** ANOVA for selected variables

Factor	*p*	Groups	Normal subjects		Patients	
			Men	Women	Men	Women
Samples (subjects)			13 (10)	13 (8)	18 (17)	25 (17)
Age	**<0.001**	**P > N**	38 ± 4	44 ± 4	54 ± 4	54 ± 4
Creatinine	0.038	M > F	11 ± 1	6 ± 1	8 ± 1	7.4 ± 0.9
Phosphate	NS	—	2.1 ± 0.2	2.3 ± 0.2	2.1 ± 0.1	2.0 ± 0.1
Sulfate	NS	—	3.8 ± 0.4	3.8 ± 0.4	3.09 ± 0.36	3.0 ± 0.4
Urine organic anion*	NS	—	2.8 ± 0.4	3.2 ± 0.3	3.3 ± 0.3	3.8 ± 0.2
GI anion	NS	—	2.6 ± 0.8	3.0 ± 0.8	2.9 ± 0.6	2.1 ± 0.6
Acid production**	NS	—	6.6 ± 0.6	7.1 ± 0.6	6.3 ± 0.5	7 ± 0.4
Ammonia	NS	—	2.4 ± 0.4	2.3 ± 0.4	2.0 ± 0.3	2.9 ± 0.3
pH	0.029	M > F	6.4 ± 0.2	5.9 ± 0.1	6.2 ± 0.1	6.0 ± 0.1
Titratable acid	NS	—	1.0 ± 0.1	1.4 ± 0.1	1.2 ± 0.1	1.3 ± 0.1
Acid excretion	NS	—	3.7 ± 0.5	3.7 ± 0.5	3.2 ± 0.4	4.2 ± 0.3
Acid balance	NS	—	0.6 ± 0.6	0.4 ± 0.5	0.2 ± 0.4	0.7 ± 0.4

Note

All factors are urine concentrations (mEq/mmol urine creatinine) except sample number, age (years), pH, and urine creatinine itself (mmol/L). Acid production is urine sulfate + titrated anion; acid excretion is urine ammonia + TA; acid balance is [(urine sulfate + anion) − (urine acid excretion + GI anion)]. All ANOVA used age as a covariate; ANOVA *p* values refer to the significance of the ANOVA, which arose from effects of patients versus normals for age, and male (M) versus female (F) for urine creatinine and pH.

* Varies directly with age, *p* < 0.015.

** *p* = 0.03.

Bold indicates P ‐ Patients and N ‐ Normal subjects.

### 3.2 Effects of sulfate, urine organic anion, and GI anion on AE

**FIGURE 1:**
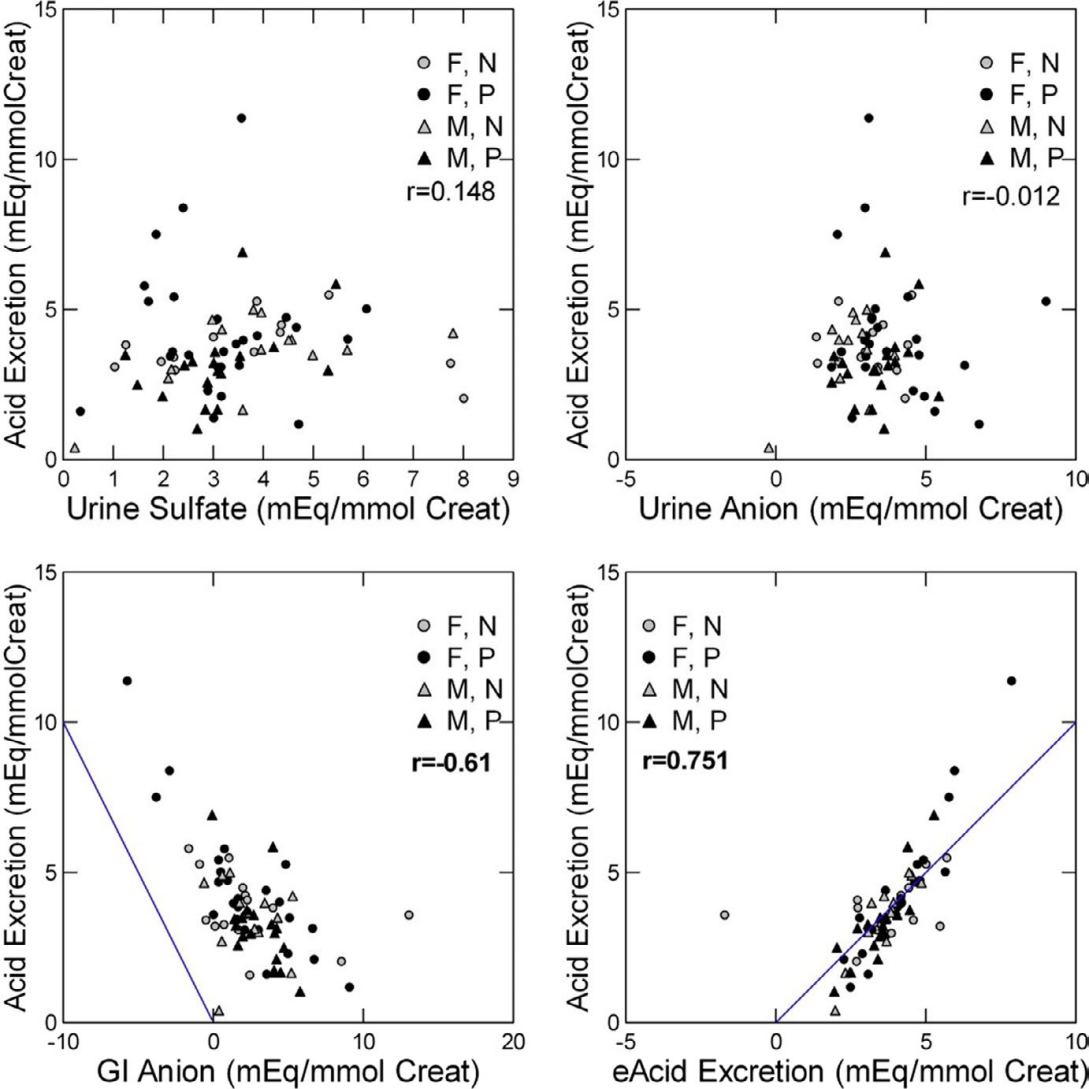
Relationship between acid excretion and components of acid production. Acid excretion correlated poorly with urine sulfate (upper left panel) and urine organic anion (upper right panel) but correlated inversely with GI anion (lower left panel). Measured acid excretion correlated well with acid excretion estimated using the regression equation in Table 2 (AE/CR) (lower right panel). Pearson *r* values give the correlation between the two variables. Bold, *p* < 0.01

Neither sulfate nor urine organic anion correlated individually with AE (Figure 1, upper panels). AE and GI anion correlated negatively (*p* < 0.001) with an appreciable offset from the line of identity (Figure 1, lower left panel). This offset would be expected if GI anion overestimates alkali production.

When GI anion, sulfate, urine organic anion, age, sex, and subject type (normal vs. patient) were combined in a single GLM, only urine sulfate, urine organic anion, and GI anion entered the model (Table 2). About half of the variation in AE remained unexplained (*R* ^2^ = 0.54). All three variables had high tolerances (0.77, 0.95, and 0.74 for urine organic anion, sulfate, and GI anion, respectively), indicating strong independent effects and lack of co‐linearity. However, within the model, urine organic anion and GI anion had a significant correlation (0.48), and perhaps for this reason, neither had as high a tolerance as did urine sulfate. Values for estimated acid excretion (eAcid Excretion) using the regression equation (Table 2, AE/CR) correlated strongly (*r* = 0.751, *p* < 0.001) with AE (Figure 1, lower right panel). Of note, the points cluster closely around the line of identity with no appreciable offset.

**TABLE 2:** General linear models of selected variables

	AE/CR	NH_4_/CR	TA/CR	pH	Phosphate/CR
Constant	**2.2**	**1.4**	**0.71**	**6.27**	**1.26**
Sulfate/CR	**0.34 ± 0.09**	**0.23 ± 0.08**	**0.10 ± 0.04**	**−0.11 ± 0.04**	0.09 ± 0.04**
Anion/CR	**0.49 ± 0.12**	**0.41 ± 0.10**	—	NS	**0.16 ± 0.05**
GI anion/CR	**−0.51 ± 0.06**	**−0.41 ± 0.05**	**−0.08 ± 0.02**	**0.09 ± 0.02**	—
Age/10 years	—	—	0.08±0.03*	NS	
Sex	—	—	—	**−0.15 ± 0.06**	—
Adj mult *R* ^2^	0.54	0.50	0.34	0.44	0.19

Note

All values are mEq/mmol creatinine. Values are coefficients for the interaction between the dependent variables (columns) and their covariates (rows). Bold, *p* ≤ 0.005.

Abbreviations: —, variable did not enter model; AE/CR, acid excretion; anion, urine organic anion; GI anion, urine GI anion; NH_4_/CR, ammonium ion; NS, variable entered model, *p* > 0.05; pH, urine pH; phosphate, urine phosphate; sulfate, urine sulfate; TA/CR, titratable acidity.

* *p* = 0.015.

** *p* = 0.026.

### 3.3 Effects of sulfate, urine organic anion, and GI anion on ammonia

**FIGURE 2:**
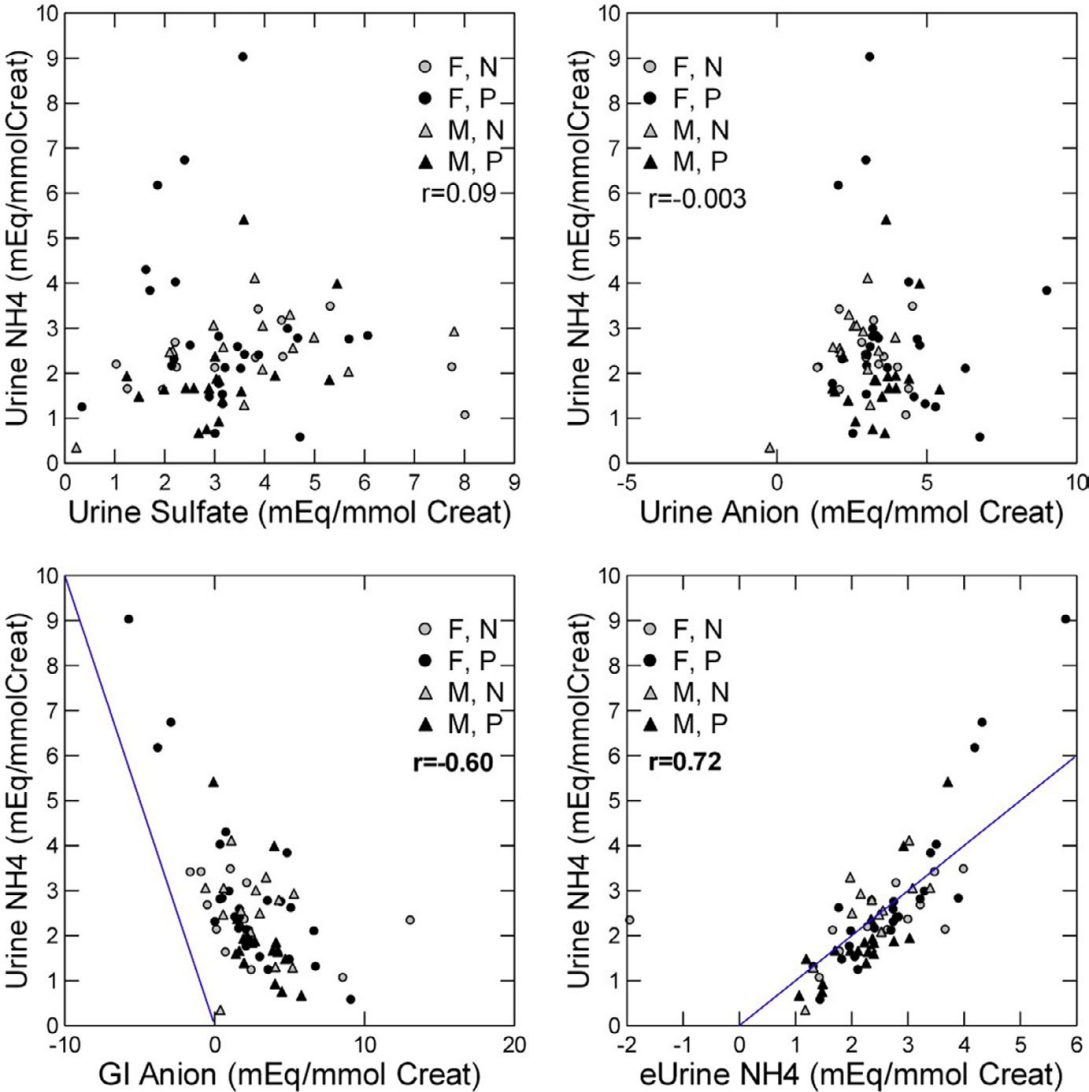
Relationship between urine ammonia and components of acid production. Urine ammonia correlated poorly with urine sulfate (upper left panel) and urine organic anion (upper right panel) and correlated inversely with GI anion (lower left panel). Measured urine ammonia correlated well with ammonia estimated using the regression equation in Table 2 (NH_4_/CR) (lower right panel). Pearson *r* values give the correlation between the two variables. Bold, *p* < 0.01

Individual correlations between urine ammonia and either urine sulfate or urine organic anion (Figure 2, upper panels) were negligible. That for GI anion was significant and inverse, as for AE itself (*p* < 0.001). Once again, points are strongly offset to the right.

In a GLM focused on urine ammonia as dependent variable, and using the same covariates as for AE, only urine sulfate, urine organic anion, and GI anion entered the model (Table 2, NH_4_/CR). Within the GLM, all three terms had high tolerances and low variable inflation factor (VIF​) (0.77, 0.95, and 0.74 [tolerance], and 1.3, 1.1, and 1.3 [VIF] for urine organic anion, sulfate, and GI anion, respectively). Notably, the coefficient for sulfate was about half that for GI anion and urine organic anion, and the former had a negative direction. Estimated ammonia from the model correlated strongly with measured urine ammonia (Figure 2, lower right panel), but the model captures only about half of the available variation (*R* ^2^ = 0.50).

### 3.4 Effects of urine sulfate, GI anion, and urine organic anion on TA

**FIGURE 3:**
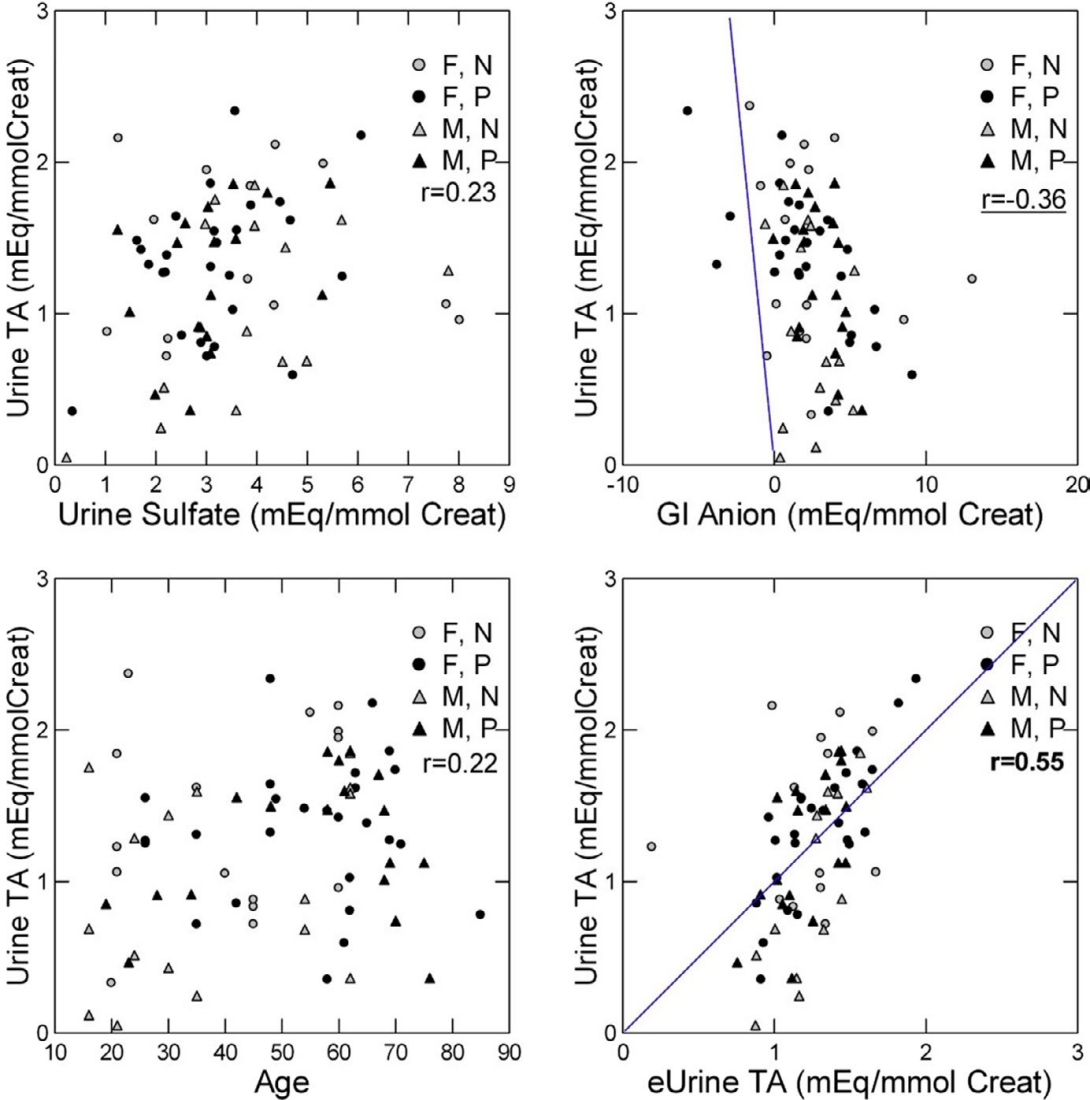
Relationship between urine titratable acidity and components of acid production and age. Urine TA correlated modestly with urine sulfate (upper left panel) and significantly with GI anion (upper right panel). There was also a modest relationship between TA and age (lower left panel). TA calculated conventionally correlated moderately well with TA estimated using the regression equation in Table 2 (TA/CR) (lower right panel). Pearson *r* values give the correlation between the two variables. Bold, *p* < 0.01; underline, *p* < 0.05

TA correlated poorly (*p* = 0.049) with sulfate (Figure 3, upper left panel) but significantly with GI anion (*p* = 0.001) (Figure 3, upper right panel). The correlation with urine organic anion was null and not graphed. Because TA was related to age in the GLM (Table 2), we graphed the individual relationship (Figure 3, lower left panel).

In a GLM with TA as dependent and using the same covariates as for AE, the final model included only GI anion, urine sulfate, and age and accounted for only 34% of the available variance (Table 2). Estimated TA from the coefficients of the model was reasonably well correlated to TA (Figure 3, lower right panel), but overall results are less impressive than for ammonia as judged from the lower individual value for *r* (0.55, *p* < 0.001).

### 3.5 Effects of urine sulfate, GI anion, and urine organic anion on urine pH and phosphate

#### 3.5.1 Urine pH

**FIGURE 4:**
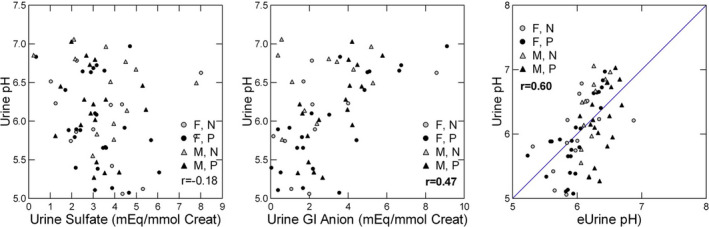
Relationship between urine pH and components of acid production. Urine pH has a significant inverse relationship with urine sulfate (left panel) and a stronger positive relationship with GI anion (middle panel). Measured urine pH correlates well with urine pH estimated from the GLM in Table 2 (right panel). Pearson *r* values give the correlation between the two variables. Bold, *p* < 0.01

Although we calculate TA from urine pH titration of phosphate, creatinine, and urate, the vast majority of the effect arises from phosphate titration, so the effects of GI anion, urine organic anion, and sulfate on pH and phosphate are of most importance to understanding their effects on TA. Urine pH has a weak but visible inverse relationship to urine sulfate (*p* = 0.005) (Figure 4, left panel) and a more robust and significant relationship to GI anion (*p* = 0.0003) (Figure 4, middle panel). In a GLM with urine pH as dependent and the same covariates as for AE, sulfate and GI anion were included, but not urine organic anion (Table 2). Sex had a significant effect (Table 2) which in this set of subjects shows a lower pH in females. pH estimated by sex from the coefficients of the model correlated significantly with urine pH (Figure 4, right panel).

#### 3.5.2 Urine phosphate

In a GLM with urine phosphate as dependent and the same covariates as for AE, urine organic anion and sulfate entered (Table 2), but the model accounted for almost none of the variance (19%). Therefore, the relationships between TA and GI anion and sulfate arise mainly from their effects on urine pH.

## 4 DISCUSSION

### 4.1 Acid and ammonia excretion

#### 4.1.1 GI anion, urine organic anion, and sulfate drive acid and ammonia excretion

It appears that AE responds to the net input of these three components of acid production and that the three act independently of one another (Table 2), based on the fact that tolerances for the three components are high in our models. Sulfate derived from metabolism of sulfur in methionine and cysteine and endogenous acid production each contribute significantly to acid production in our subjects. GI anion has a negative coefficient, consistent with the understanding that it represents alkali ingested from food. Age, sex, and patient type do not enter the model in this set of data, although larger studies are required to fully explore this question. The same relationships hold for ammonia.

#### 4.1.2 GI anion and urine organic anion may not act entirely as acids or bases

Given the assumption that AE responds to the interactions of GI anion, urine organic anion, and sulfate for the maintenance of acid–base balance, our analysis suggests that the interaction is complex. The coefficients within the models are rather modest, in the range of 0.3–0.5 mEq of acid or ammonia excretion per unit change of sulfate, urine organic anion, or GI anion. The modest magnitude of the partial coefficients is compatible with incomplete metabolism of food anions and consequent titration of unmetabolized food anions. The poor correlation between AE and urine organic anion (Figure 1, upper right panel) suggests that the identity of these anions may vary, and some may not represent acid generation, but instead ingestion of unmetabolized anion, as suggested by others (Lemann et al., 2003). Our present experiments cannot take this problem further but suggest a need for new experimental designs that can quantify the fraction of food anion metabolized, and the nature of the organic anions that are present in urine.

### 4.2 Urine sulfate and GI anion but not urine organic anion affect urine pH and TA

Total urine organic anion had no correlation with urine pH or TA, either individually or within our models as evidenced by its failure to achieve a sufficiently low *p* value to enter via the stepping algorithms (Section 2). Metabolically produced acids (reported as urine organic anion) have a robust association with urine ammonia and AE, and thus, their failure to affect urine pH may be surprising, but this has been reported previously for total urine organic anion (Lemann et al., 2003). In fig. 5A of Lemann et al. (2003), as urine pH varies in humans given NH_4_Cl or potassium bicarbonate, no change in organic anion excretion occurs. If we invert that graph, it means that changes in organic anion excretion do not correlate with changes in urine pH. The fraction of urine organic anions measured between the pH of the urine and pH 7.4 are excreted as free organic acids, with their protons, and therefore do not contribute to acid production, while the fraction measured between pH 2.7 and urine pH—80% or more of the total urine organic anion—are excreted as anions and are a component of acid production. This latter component has been shown by others to vary with urine pH (Lemann et al., 2003), and future studies should take this into account in assessing total acid production.

Why sulfate ion but not titrated urine anion correlates inversely with urine pH is not clear. Experimental sulfate infusion strikingly lowers urine pH (Coe & Korty, 1967; Lemann & Relman, 1959), suggesting some effects not presently fully understood.

GI anion, which is positively correlated with urine pH, estimates bicarbonate production, even if not quantitatively, and urine pH is related to variations in serum bicarbonate. In addition, secretin, a duodenal hormone that increases pancreatic exocrine function, is linked to bicarbonate secretion in the beta intercalated cells of the collecting duct (Berg et al., 2020), and this may link increased gut alkali absorption to an increase in urine pH.

### 4.3 Relationship between urine organic anion and AE in prior studies

Few prior studies (Table 3) have examined the role of urine organic anion in total AE. Balance studies done mostly in healthy young males on constant diets show stable organic anion excretion that is not altered by exogenous administration of acid or alkali (Adams et al., 1979; Dominguez et al., 1976; Lemann et al., ,^1965^, ^1966^, ^1989^, ^2003^; Lemann & Relman, 1959; Lennon et al., 1966; Relman et al., 1961). In these studies, sulfate excretion and organic anion contributed about equally to acid production, but only sulfate was altered by diet. They did not examine the overall relationships between the three facets of acid production and net AE, and the studies were not powered to investigate differences in organic anion production due to age or sex. Ours is the first study to show that anion excretion increases with age.

**TABLE 3:** Previous studies using urine organic anion

Reference	Balance study	Subjects	Experimental design/intervention	Urine organic anion levels (mEq/day)
Lemann et al. JCI 38:2215, 1959	Yes	3 M	Liquid formula diet Control, methionine	Shown for 1 subject at multiple time points
Relman et al. JCI 40:1621 1961	Yes	9 M	Liquid formula diet Control, NaHCO_3_, Na orthophosphate, acetazolamide	Not reported individually but summed with sulfate
Lemann et al. JCI 44:507, 1965	Yes	14 M	Liquid formula diet Control, NH_4_Cl, NaHCO_3_	Shown for 2 subjects, otherwise summed with sulfate
Lemann et al. JCI 45:1608 1966	Yes	5 M	Whole food diet Control, NH_4_Cl	Not reported individually but summed with SO_4_ (Reported in AJP Renal Physiol F811–F832, 2003)
Lennon et al. JCI 45:1601 1966	Yes	14 M	Liquid formula diet, two whole food diets	Not reported individually, but summed with SO_4_
Litzow et al. JCI 46:280, 1967	Yes	7 (3 F, 4 M) with CKD	Whole food diets Control, NaHCO_3_	36 ± 7 and 44 ± 9 (control and alkali; *p* = 0.013) (Reported in AJP Renal Physiol F811–F832, 2003)
Dominguez et al. JCEM 43:1047, 1976	Yes, and ad lib	6 (3 F, 3 M)	Control, NH_4_Cl	(Reported in AJP Renal Physiol F811–F832, 2003)
Adams et al. Calcif Tissue Int 27:233, 1979	Yes	7	Control, NH_4_Cl (3), protein (4)	(Reported in AJP Renal Physiol F811–F832, 2003)
Lemann et al. KI 35 1989	Yes	9 M	Whole food diets Control, KHCO_3_, NaHCO_3_	35 ± 6, 38 ± 7, and 38 ± 9 (control, KHCO_3_, and NaHCO_3_, respectively)
Uribarri et al. KI 47:624, 1995	No	32 (19 F, 13 M) with CKD, 8 N	24‐h urines on ad lib diets	45 ± 5.5, 45 ± 4, and 41.5 ± 3 CKD normal bicarbonate, CKD low bicarbonate, and N

### 4.4 Urine organic anion and acid production increase with age

In our ANOVA models, we used age as a covariate and noticed that urine organic anion and total acid production correlated positively with age. However, as the stone formers were older than the control subjects, we cannot rule out an association of organic anion production with stone formation. Our present data set does not permit us a further exploration of this finding. However, in our past work on the fall of urine pH with age in stone formers (Menezes et al., 2019), we proposed that one possibility might be an increase in acid load with age. Possibly, increase of acid production and urine organic anion excretion is a consequence of aging, a matter for future experiments.

### 4.5 Our models account for only half of AE

In the model, the constant of 2.2 mEq/mmol creatinine (Table 2) and the multiple *R* ^2^ value for the GLM on AE of 0.54 imply that a significant component of AE is unaccounted for.

#### 4.5.1 Lack of stable diet

Given that GI anion, urine organic anion, and sulfate undoubtably in aggregate are the main acid load, it might appear strange that nearly one half of the variation in AE is not accounted for. We suspect this is because we made no effort to stabilize diet. When AE is deliberately varied, urine ammonia adjusts over several days (Lemann et al., 1966). Under our conditions, urine ammonia presumably reflects the integrated average of several prior days, whereas our estimates arise from 1 day. Models using data sets from subjects fully adapted to one diet may well account for more of the variation in AE. Nonetheless, while diet affects acid production through sulfate and GI anion, there is no current evidence that diet affects endogenous acid production.

Our study design did not include food diaries. Our primary objective was to identify the independent effects of sulfate, titrated anion, and GI anion on renal AE. The present results suggest that there is value in a more extensive experiment using controlled diet conditions. We predict that a higher fraction of variation will be captured by our regressions, and comparisons between the sexes and patient groups will be of scientific value. We also expect that with controlled diets we will find a higher urine pH in women versus men as has been found in other studies. The present finding of a lower urine pH in women is presumably because of lack of diet control.

#### 4.5.2 Lack of urine TCO_2_

Another problem is our lack of urine total CO_2_. This removes variabilities of net AE—which is the true response to acid load—due to fluctuating urine bicarbonate. As a test, we redid the GLM for AE limiting ourselves to cases where urine pH <6.2. Below this pH, urine TCO_2_ is known to be very low (Lemann et al., 2003). The value for *R* ^2^ was higher (0.74), but we had only 37 measurement sets. Of perhaps some interest, the coefficient for sulfate fell to 0.21, and those for urine anion and GI anion rose to 0.81 and −0.76, respectively. Another important issue related to urine TCO_2_ is that without it our GI anion cannot be corrected for bicarbonate and is therefore too high when pH is above 6.2.

## 5 SUMMARY

This work highlights the importance of understanding the contribution of organic anion excretion to acid production and acid balance, in aging as well as in disease states. Future studies with controlled diets, particularly in older subjects, can progress this work. The nature and amount of the urine organic anions may reflect changes in metabolism with age. The effect of systemic disease, such as chronic kidney disease (CKD), on organic anion excretion is also not well studied. The two studies in the literature (Litzow et al., 1967; Uribarri et al., 1995) suggest that alkali administration may have different effects in patients with CKD compared with normal subjects. In addition to amount of titrated organic anion, the nature of these anions and their sources will be of interest in understanding the metabolome of disease states.

## CONFLICT OF INTEREST

The authors declare no conflict of interest.

## AUTHOR CONTRIBUTIONS

Huo, Li, McKay, and Hoke: method development, data acquisition, and analysis; Worcester: study design and preparation of manuscript; Coe: study design, data analysis, and preparation of manuscript. All authors approved the final version of the manuscript.
